# Effects of public and private health insurance on medical service utilization in the National Health Insurance System: National panel study in the Republic of Korea

**DOI:** 10.1186/s12913-016-1746-2

**Published:** 2016-09-21

**Authors:** Minsung Sohn, Minsoo Jung

**Affiliations:** 1 Department of Public Health Sciences, Graduate School of Korea University, Seoul, South Korea; 2 BK21PLUS Program in Embodiment: Health-Society Interaction, Korea University, Seoul, South Korea; 3 Department of Health Science, Dongduk Women’s University, 23-1 Wolgok-dong, Seongbuk-gu, Seoul, 136-714 South Korea

**Keywords:** National health insurance, Private health insurance, Medical service utilization, Moral hazard, South Korea

## Abstract

**Background:**

Moral hazard or utilization hazard refers to the phenomenon during which patients overuse medical services under national health insurance (NHI) because the services are free or the patients are required to pay only a portion of the utilization costs. The aim of this study is to investigate how NHI and private health insurance (PHI) systems influence increases in health care utilization rates.

**Methods:**

We designed a longitudinal study to examine the utilization of healthcare services between those insured with NHI or PHI and uninsured Koreans using nationally representative four-year panel data from 13,798 participants. This study was conducted using hierarchical multivariate Poisson regression analyses in which covariates and interaction terms are applied after adjusting for the heterogeneous treatment effect.

**Results:**

After adjusting covariates including disease status, lower income Koreans who were covered by medical aid were respectively 2.26 and 1.23 times more likely to receive inpatient care and outpatient care than those who were covered by NHI. When the interaction term of type of insurance was included in the model, those were covered by both medical aid and PHI were respectively 2.38 and 1.25 times more likely to receive inpatient care and outpatient care than those who were covered by only NHI.

**Conclusions:**

The moral hazard behind insurance membership, depending on how NHI maintains policies to confer benefits, may give rise to differences in medical utilization. This phenomenon must be closely monitored to find a way to reform NHI when the rights of medical service consumers are solidified through PHI.

## Background

**Fig. 1:**
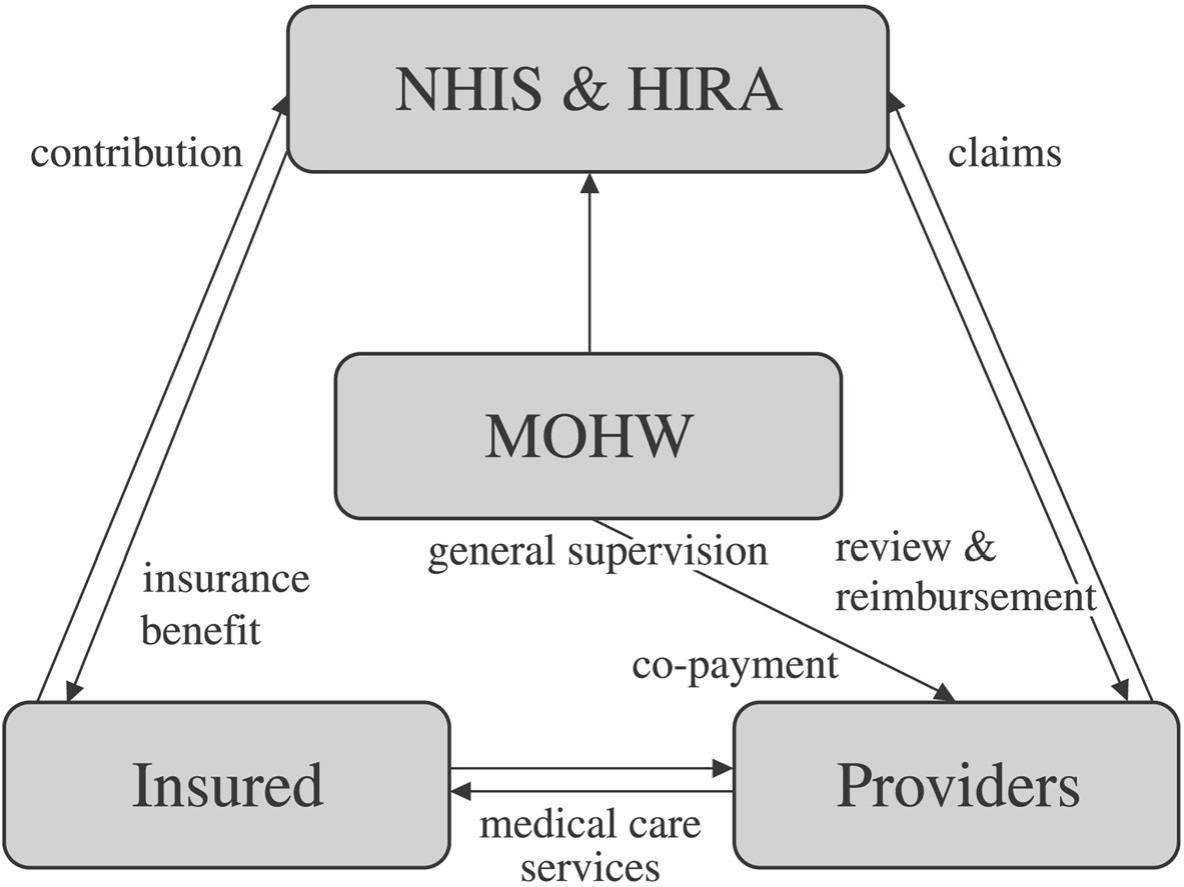
Relations among major parties of Korean NHI. NHIS: National Health Insurance Service. HIRA: Health Insurance Review & Assessment Service. MOHW: Ministry of Health and Welfare

**Fig. 2:**
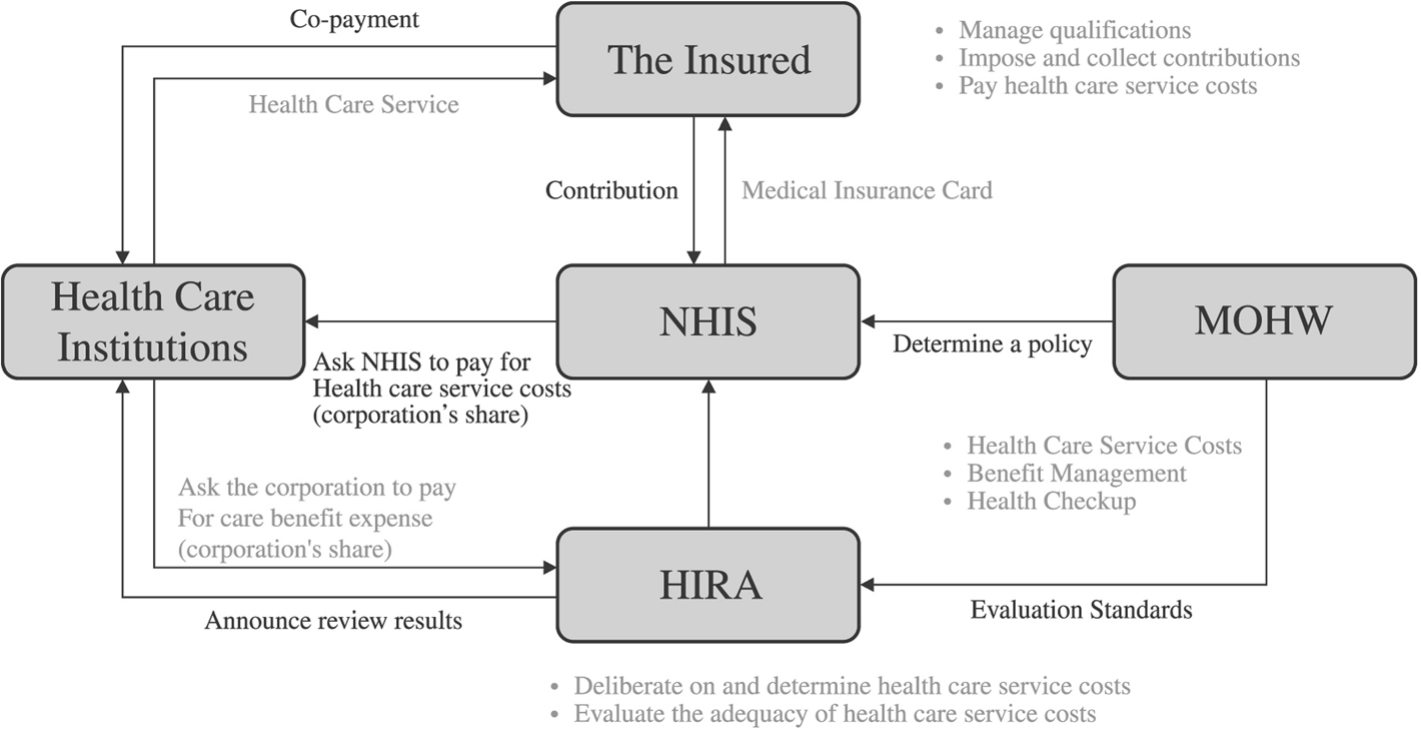
NHI system in South Korea. NHIS: National Health Insurance Service. HIRA: Health Insurance Review & Assessment Service. MOHW: Ministry of Health and Welfare. Retrieved from http://www.nhis.or.kr/static/html/wbd/g/a/wbdga0401.html. (November 6, 2015)

The health care system in the Republic of Korea is implemented under the National Health Insurance (NHI) program, which is compulsory by law and is a universal social insurance program that covers the entire population (Fig. 1). This system is operated by the NHI Service (NHIS) under the supervision of the Korean government. The single insurer, termed the NHIS, promotes the population’s health and strives to improve social security by providing necessary health care services pertaining to disease, injury, birth and death (Fig. 2). It also operates a free medical aid program as a form of public assistance for those in the low-income bracket, for whom the application of social insurance is difficult through NHI [1–3]. This implies that medical aid in Korea plays an important role as a social security system for those in the lower income brackets, who pass a means test and who are subsidized by local governments and not by the NHI system [1–4]. Patriots and veterans who receive benefits as a person of national merit are also included in the medical aid group. The proportion of medical aid beneficiaries is about 3 % of the total population in South Korea.

In spite of implementing the NHI system, the Korean government is contemplating the expansion of private health insurance (PHI) in the interest of patient sovereignty [3, 5, 6]. According to categories of the health security system used by a previous study, the Korean system may be primarily categorized as corporatism supplemented by universalism [7]. The basic reasoning behind this categorization is that health insurance financing is based on individual insurance premiums, while benefits such as welfare services are linked to income [1, 8]. However, it is dubious as to whether Korea’s NHI system is effective. When NHI coverage is calculated as the ratio of public resources to individual medical expenditures, the resulting rate of 57.2 % is far less than the Organization for Economic Cooperation and Development (OECD) average of 71.3 % [9]. The situation of Korea is thus identical to that of countries such as Portugal, which must rely on PHI because the income range is unsecured and limited [10]. The individual purchase of PHI is part of liberalism; the nation’s social security benefits are not sufficient such that healthcare issues are primarily the responsibility of the individual, whereas the supply of medical services is the responsibility of the private sector [4, 11]. Of course, many countries utilizing a NHI system endeavor to incorporate the merits of liberalism by introducing PHI, and Korea is not the only country to do so [6]. However, the consequences of doing so are rarely discussed. This needs to be discussed by highlighting how the type of health insurance affects the healthcare service utilization of patients.

Moral hazard (also known as utilization hazard) is a model based on behavioral economics. According to basic economic theory, health insurance coverage may cause a reduction in prevention activities, as it lowers the cost of medical care and thereby reduces the financial and health consequences of illness [12]. This refers to the phenomenon by which, unlike in the usual market where, consumers pay the full price when purchasing goods or services, patients overuse medical services under NHI because they use medical services free of charge or pay only a portion of the utilization costs [13]. In other words, given that insurance companies or the state pays in part or in whole for their services, these insurance subscribers have a tendency to use medical services more than is necessary or to be negligent in terms of health promotion [14]. Just as drivers who have usually driven safely are likely to engage in reckless driving after subscribing to automobile insurance, people who subscribe to NHI or PHI have a tendency to increase the quantity of their medical utilization within the scope of coverage. Regardless of whether it is NHI, which is compulsory, or PHI, which is purchased voluntarily, once individuals benefit from having insurance, there is the possibility that the phenomenon known as moral hazard can arise [15–17]. Although the policy of user fees is established to prevent the overuse of medical services, it is not possible to make out-of-pocket payment amounts excessively high to strengthen the coverage of NHI. Moreover, medical aid beneficiaries are exempt from paying user fees. In addition, higher income groups, not satisfied with limited coverage and the low quality of service, emphasize patient sovereignty and claim that individuals should be encouraged to purchase various types of PHI [11, 18]. However, little is known about whether increased purchases of PHI will increase the utilization of medical services under the NHI system.

Although it is economically rational behavior for insurance subscribers to engage in more medical utilization from the standpoint of patients, the welfare loss decreases for the entire state [13, 19]. Consequently, most countries that adopt social insurance-type health security systems have institutionalized the cost sharing of a portion of the fees by consumers according to healthcare utilization. Through this, the aim is to suppress unnecessary healthcare utilization and to provide economic motivation so that consumers will resolve their health problems without the seeking of medical services [3, 13, 15, 20].

The Korean NHI implemented a co-payment ceiling system to prevent the abuse of medical utilization and to intensify the security efficiency of NHI, although types of user fees are deductibles, co-insurance, ceiling system, co-payment, and indemnity. The designated co-payment rate is 32.1 % on average, but is almost double the median of other OECD countries [1, 9, 21, 22]. However, medical utilization behavior then changes in the direction of not using services with less utility than out-of-pocket payment, so that even at the same prices it becomes more likely for those in the low-income strata to relinquish medical utilization [16]. Unless there is a considerably excessive demand in the medical service market, medical utilization is very likely to decrease if the user burden increases [22]. However, to further explore the current moral hazard or utilization hazard model [19], it is necessary to grasp medical service utilization behavior not according to the user burden, where variation among individuals is not large under the NHI system, but according to the type of NHI and PHI purchased.

In this respects, we focused on the interrelationship between national and private health insurance having influence on the insurer’s medical service utilization. In order to forecast the macro consequences of PHI expansion in the corporatism type, it is necessary to examine the healthcare service utilization behavior of patients by the insurance types. There are four types of health insurance subscribers in Korea’s health security system: (1) those with NHI but not PHI, (2) those with PHI but not NHI, (3) those with both NHI and PHI, and (4) those with neither NHI nor PHI (i.e., the basic livelihood security recipients). Each group may pursue healthcare utilizing behavior as a rational consumer.

The NHI promotes individuals’ healthcare utilization as much as it does the benefits of NHI, and PHI reimburses the users’ fees and uncovered health services. Thus, it is very likely that it will facilitate healthcare utilization. If the healthcare demands may fluctuate based on healthcare coverage, and not on clinical diagnosis of disease, then this is an unintended consequence that could damage financing of NHI [23]. Unlike the health security system, PHI calculates the insurance cost based on the individual’s level of risk; thus, it is more favorable for individuals with relatively lower risk levels to select PHI [24]. Therefore, if more individuals with relatively lower risk levels purchase PHI, they can become an interested party who demand the downsizing of the health security system. For individuals who benefit from both the public and private health insurance, their security is heightened and burden is decreased [18, 25]. The increase of healthcare utilization would probably occur on both sides.

Although Korea has achieved universal health coverage through its NHI scheme within such a short period, high out-of-pocket payment and limited financial protection under the current national system have induced a high demand for supplementary PHI. We examined the effect of Korea’s NHI and PHI on health care utilization. We also investigated to what extent health care utilization—such as the inpatient and outpatient care—NHI and PHI incur based on phenomena such as moral hazard.

## Methods

### Study sample

The data used for this study derived from a survey of 13,798 respondents drawn from a nationally representative longitudinal sample of the Republic of Korea who participated in the Korea Health Panel (KHP; www.khp.re.kr). The members of this panel were recruited from October 2008 to January 2011 using a dual sampling frame of the national population and housing census, and a combination of random digital dial and address-based sampling. Respondents received nominal cash incentives to participate in this survey, which were administered by the Korea Institute Health and Social Affairs in conjunction with the NHIS. The KHP data include information on each instance of medical service utilization and expenditure as well as the individual’s NHI and PHI status. The participants were required to collect all receipts of medical expenses to alleviate the problem of recall bias. The final response rate was 82.6 %. After excluding observations with missing values, we used a sample of 13,798 adults from 2008 (the first wave) as a 4-year unbalanced panel data set.

### Study design

We designed a longitudinal study to examine the utilization of healthcare services between those insured with NHI/PHI and those not insured with NHI/PHI using the 4-year panel data. We modeled stratified Poisson regression analyses by applying covariates and interaction terms after adjusting for the heterogeneous treatment effect [26]. We used Poisson regression because the utilization dependent variables are count measures with a skewed, nonparametric distribution, and therefore standard parametric approaches like linear regression are not statistically appropriate.

### Measures

#### Dependent variables

The outcome variables were the utilization of inpatient care and outpatient care, in accordance with the literature [27]. We asked about the utilization of inpatient care, including dental/oriental medicine hospitalization, intensive care ward, and one-day inpatient stays during the last year, and instances of outpatient visits, including dental/oriental medicine treatment. The responses were the total number of inpatient days and outpatient visits during the last year among users.

#### Independent variables

##### Socioeconomic status

We considered education and household income as baseline independent variables in the model in accordance with the knowledge that healthcare utilization varies by socioeconomic status [24, 28, 29]. The highest level of education completed was collapsed into the following categories: elementary school or less, middle school/associate degree, high school/associate degree, and college degree or higher. Household income included earned income, financial income, real estate income, and transfer income, which were divided by the number of family members, in accordance with the literature. The computational definition used was (ß = 1, V = 0.5). The household income was collapsed into the lowest 25 percent (1st quartile), middle 50%, and bottom 25 %, with the highest 25 % (4th quartile) as the standard.

Household income adjusted by number of members in the family was calculated as

$$ \mathrm{Y}/\left(\mathrm{A} + \mathrm{\ss}\mathrm{B}\right)\mathrm{v} $$

where Y = average monthly income, A = number of adults, B = number of non-adults, and ß and v = equalized index

##### Types of health insurance

**Fig. 3:**
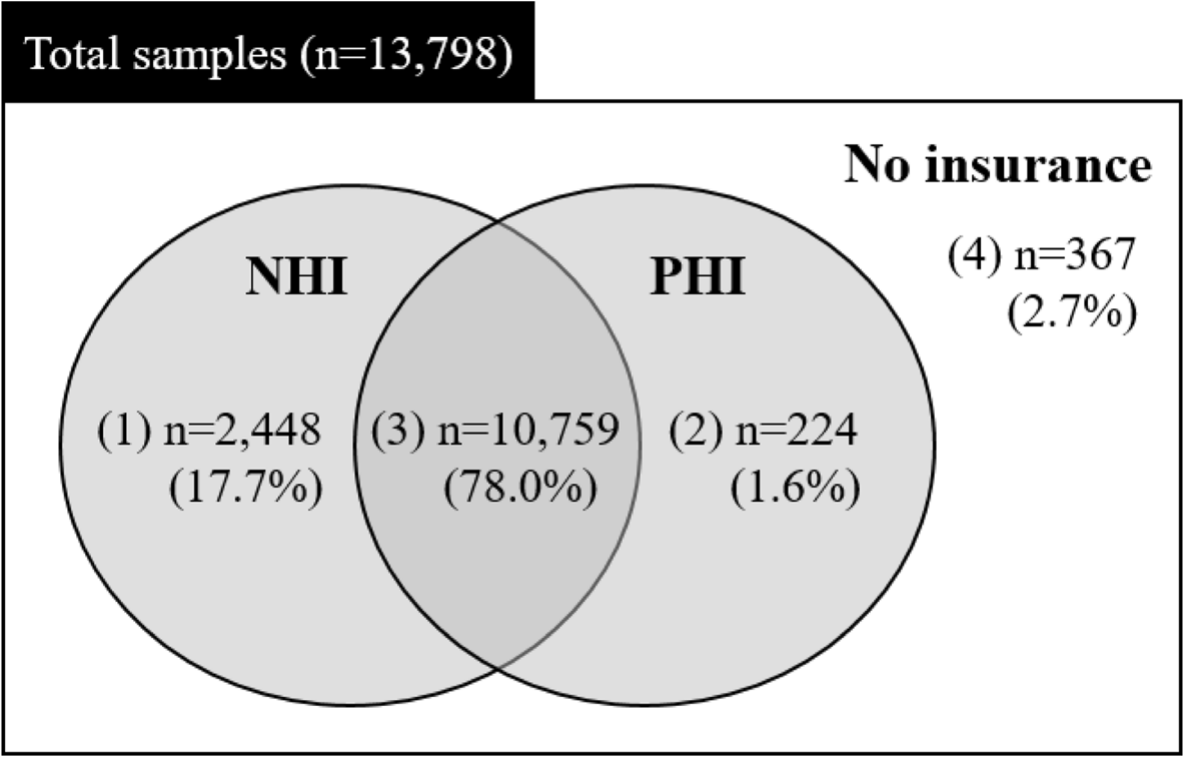
Four types of health insurance of this study (first wave, 2008). Note: NHI: National Health Insurance; PHI: Private Health Insurance

Individuals reported their NHI and PHI status, in accordance with the knowledge that the status significantly determines healthcare utilization [29, 30]. NHI status was assessed on the response to the question, “Is your healthcare covered by NHI or are you a medical aid beneficiary?” The response was yes (i.e., the entire population, including government employees with insurance bills) or no (i.e., basic livelihood security recipients or veterans/patriots). PHI status was assessed on the basis of the question, “Do you have any private health insurance or are you a beneficiary who is reimbursed for medical expenses?” with responses again categorized as yes or no. We did not use the number of PHIs purchased as a measure, since this is not significantly different from a dichotomous measure, such as yes or no [30]. We thus classified the whole sample into four categorical groups in terms of insurance coverage: (1) covered by only NHI, (2) covered by both the medical aid and PHI, (3) covered by both NHI and PHI, and (4) covered by only the medical aid program (Fig. 3).

#### Covariates

We considered covariates in accordance with the previous literature [17, 31]. Respondents were queried for sociodemographic characteristics of age, gender, chronic diseases, and marital status.

### Statistical analyses

We modeled hierarchical multivariable Poisson regression of the data to examine the effects of PHI on the utilization of inpatient and outpatient care in the NHI system, controlling for potential covariates like socioeconomic status. First, descriptive statistics and frequencies were run for all variables. Second, univariate chi-square analyses were run for each predictor variable to identify factors that were associated with the utilization of inpatient and outpatient care. Finally, we ran Poisson regression models by types of healthcare utilization with increasing significance values and retaining covariates, until we reached a model where all additional predictors were significant at *p* < 0.05. Statistical analyses were conducted by using STATA v. 12.0 software (STATA, College Station, TX).

## Results

### General sample characteristics

As detailed in Table 1, 52.3 % of the individuals were women, 26.3 % were 60 years or older, and 72.2 % were currently married. Most had a college degree or higher (34.9 %). Almost all (95.7 %) had their healthcare services covered by NHI, but 4.3 % who were below the poverty line were covered by the medical aid. 79.6 % received benefits from their own PHI, but 20.4 % had not purchased PHI. The number of days receiving inpatient care per year on average was 0.13 (SD = 0.5), ranging from 0 to 12, and the number of visits receiving outpatient care per year on average was 11.91 (SD = 19.14), ranging from 0 to 308 during the first year.

**Table 1:** General sample characteristics

	1^st^ Wave, 2008 (*n* = 13,798)		2^nd^ Wave, 2009 (*n* = 14,222)		3^rd^ Wave, 2010 (*n* = 13,336)		4^th^ Wave, 2011 (*n* = 12,347)	
	*N*	(%)	*N*	(%)	*N*	(%)	*N*	(%)
Gender								
Men	6,584	(47.7)	6,800	(47.8)	6,377	(47.8)	5,894	(47.7)
Women	7,214	(52.3)	7,422	(52.2)	6,959	(52.2)	6,453	(52.3)
Age								
20–29	1,943	(14.1)	2,006	(14.1)	1,851	(13.9)	1,407	(11.4)
30–39	2,893	(21.0)	2,828	(19.9)	2,528	(19.0)	2,138	(17.3)
40–49	2,953	(21.4)	3,071	(21.6)	2,855	(21.4)	2,715	(22.0)
50–59	2,381	(17.3)	2,440	(17.2)	2,277	(17.1)	2,235	(18.1)
60 or older	3,628	(26.3)	3,877	(27.3)	3,825	(28.7)	3,852	(31.2)
Chronic disease								
Yes	6,474	(46.9)	7,642	(53.7)	7,626	(57.2)	7,630	(61.8)
No	7,324	(53.1)	6,580	(46.3)	5,710	(42.8)	4,717	(38.2)
Marital status								
Single	2,300	(16.7)	2,569	(18.1)	2,383	(17.9)	2,043	(16.6)
Married couple	9,955	(72.2)	10,034	(70.6)	9,407	(70.5)	8,805	(71.3)
Divorced or Separated	392	(2.8)	410	(2.9)	397	(3.0)	386	(3.1)
Separation by death	1,151	(8.3)	1,209	(8.5)	1,149	(8.6)	1,113	(9.0)
Education								
Elementary School or less	2,955	(21.4)	2,996	(21.1)	2,868	(21.5)	2,718	(22.0)
Middle School to associate	1,565	(11.3)	1,576	(11.1)	1,487	(11.2)	1,419	(11.5)
High School to associate	4,462	(32.3)	4,519	(31.8)	4,206	(31.5)	3,892	(31.5)
College Degree or higher	4,816	(34.9)	5,131	(36.1)	4,775	(35.8)	4,318	(35.0)
Household income								
4^st^ quartile (highest)	3,550	(25.7)	3,558	(25.0)	3,336	(25.0)	3,364	(27.3)
3^st^ quartile	3,471	(25.2)	3,556	(25.0)	3,354	(25.2)	2,944	(23.8)
2^st^ quartile	3,390	(24.6)	3,559	(25.0)	3,319	(24.9)	2,934	(23.8)
1^st^ quartile (lowest)	3,387	(24.6)	3,549	(25.0)	3,327	(25.0)	3,105	(25.2)
Healthcare covered by NHI								
Yes	13,207	(95.7)	13,563	(95.4)	12,642	(94.8)	11,732	(95.0)
No (medical aid)	591	(4.3)	659	(4.6)	694	(5.2)	615	(5.0)
Purchase of PHI								
Yes	10,983	(79.6)	11,612	(81.7)	10,537	(79.0)	9,973	(80.8)
No	2,815	(20.4)	2,610	(18.4)	2,799	(21.0)	2,374	(19.2)
The number of days receiving inpatient care for a year	0.13 ± 0.50 (0–12)		0.16 ± 0.60 (0–22)		0.19 ± 0.68 (0–24)		0.19 ± 0.79 (0–41)	
The number of visits receiving outpatient care for a year	11.91 ± 19.14 (0–308)		13.05 ± 21.15 (0–455)		14.60 ± 22.59 (0–350)		15.97 ± 24.49 (0–351)	

*Note*: *NHI* National Health Insurance, *PHI* Private Health Insurance

The number of days receiving inpatient care and the number of visits receiving outpatient care are presented as mean ± SD (range)

### Differences between the utilization of inpatient and outpatient care/non-utilization of inpatient and outpatient care groups

As shown in Table 2, compared to the non-utilization of inpatient care group, the utilization of inpatient care group had more participants who were women (*p* < 0.001), 60 years or older (*p* < 0.001), lower educational attainment (*p* < 0.001), and more individuals in the highest quartile range of household income (*p* < 0.001). Regarding health insurance, there was a gap between the NHI-covered group and the medical aid beneficiaries in terms of utilization of inpatient care (*p* < 0.001). There was also a gap between subscribers and non-subscribers to PHI (*p* < 0.001). Almost similar differences were shown between the utilization of inpatient care and the non-utilization of inpatient care groups. The utilization of the outpatient care group consisted of more participants with relatively higher educational attainment (*p* < 0.001). There was a significant difference between the utilization of inpatient and outpatient care and the non-utilization of inpatient and outpatient care groups by types of health insurance coverage (*p* < 0.001).

**Table 2:** Differences between the utilization of inpatient and outpatient care/non-utilization of inpatient and outpatient care groups, 2008 (*n* = 13,798)

	Inpatient care					Outpatient care				
	Utilization group		Non-utilization group		Chi square	Utilization group		Non-utilization group		Chi square
	(*n* = 818)		(*n* = 12,980)		*p*-value	(*n* = 9,377)		(*n* = 4,421)		*p*-value
	*N*	(%)	*N*	(%)		*N*	(%)	*N*	(%)	
Gender										
Men	342	(41.8)	6,242	(48.1)	<0.001	3,813	(40.7)	2,771	(62.7)	<0.001
Women	476	(58.2)	6,738	(51.9)		5,564	(59.3)	1,650	(37.3)	
Age										
20–29	72	(8.8)	1,871	(14.4)	<0.001	830	(8.9)	1,113	(25.1)	<0.001
30–39	140	(17.1)	2,753	(21.2)		1,667	(17.8)	1,226	(27.7)	
40–49	125	(15.3)	2,828	(21.8)		1,869	(19.8)	1,084	(24.5)	
50–59	125	(15.3)	2,256	(17.4)		1,824	(19.5)	557	(12.7)	
60 or older	356	(43.5)	3,272	(25.2)		3,187	(34.0)	441	(10.0)	
Chronic disease										
Yes	600	(73.4)	5,874	(45.3)	<0.001	6,088	(64.9)	386	(8.7)	<0.001
No	218	(26.6)	7,106	(54.7)		3,289	(35.1)	4,035	(91.3)	
Marital status										
Single	55	(6.7)	2,245	(17.3)	<0.001	859	(9.2)	1,441	(32.6)	<0.001
Married couple	619	(75.7)	9,336	(71.9)		7,207	(76.8)	2,748	(61.1)	
Divorced or Separated	40	(4.9)	352	(2.7)		295	(3.2)	97	(2.2)	
Separation by death	104	(12.7)	1,047	(8.1)		1,016	(10.8)	135	(3.1)	
Education										
Elementary School or less	283	(34.5)	2,672	(20.6)	<0.001	2,554	(27.2)	401	(9.1)	<0.001
Middle School to associate	101	(12.4)	1,464	(11.3)		1,257	(13.4)	308	(7.0)	
High School to associate	238	(29.1)	4,224	(32.5)		2,926	(31.2)	1,536	(34.7)	
Bachelor’s Degree or higher	196	(24.0)	4,620	(35.6)		2,640	(28.2)	2,176	(49.2)	
Household income										
4^st^ quartile (highest)	294	(35.9)	3,256	(25.1)	<0.001	2,708	(28.8)	842	(19.1)	<0.001
3^st^ quartile	185	(22.6)	3,286	(25.3)		2,269	(24.2)	1,202	(27.2)	
2^st^ quartile	175	(21.4)	3,215	(24.8)		2,161	(23.1)	1,229	(27.8)	
1^st^ quartile (lowest)	164	(20.1)	3,223	(24.8)		2,239	(23.9)	1,148	(25.9)	
Healthcare covered by NHI										
Yes	728	(89.0)	12,479	(96.1)	<0.001	8,878	(94.6)	4,329	(97.9)	<0.001
No (medical aid)	90	(11.0)	501	(3.9)		499	(5.4)	92	(2.1)	
Purchase of PHI										
Yes	605	(73.9)	10,378	(80.0)	<0.001	7,291	(77.7)	3,692	(83.5)	<0.001
No	213	(26.1)	2,602	(20.0)		2,086	(22.3)	729	(16.5)	
Type of insurance										
Covered by only NHI	157	(6.41)	2,291	(93.59)	<0.001	1,775	(72.51)	673	(27.49)	<0.001
Covered by only the medical aid	56	(15.26)	311	(84.74)		311	(84.74)	56	(15.26)	
Covered by both NHI and PHI	571	(5.31)	10,188	(94.69)		7,103	(66.02)	3,656	(33.98)	
Covered by both the medical aid and PHI	34	(15.18)	190	(84.82)		188	(83.93)	36	(16.07)	

*Note*: *NHI* National Health Insurance, *PHI* Private Health Insurance

### Influential factors pertaining to the utilization of inpatient and outpatient care among panel participants

As detailed in Table 3, after controlling for covariates, those who had completed elementary school or less were 1.36 times more likely to receive inpatient care as compared to the participants with a college degree or higher, as shown in Model II (95 % CI: 1.21–1.54). Moreover, those in the highest income bracket were 1.15 times more likely to receive inpatient care than those in the lowest income bracket (95 % CI: 1.06–1.26). When we added each of the health insurance factors in Model III after controlling for covariates, medical aid beneficiaries were 2.26 times more likely to receive inpatient care than those who were covered by NHI (95 % CI: 2.01–2.53). When we added the interaction terms in Model IV, those who were covered by both the medical aid program and PHI were 2.38 times more likely to receive inpatient care than those who were covered by NHI (95 % CI: 1.97–2.76). On the other hand, those who were covered by only medical aid had a tendency to use services less than those who were covered by both the medical aid program and PHI, and they were 2.37 times more likely to receive inpatient care than those who were covered by only NHI (95 % CI: 2.00–2.75). We also examined the effects of PHI on the utilization of outpatient care in the NHI system (Table 4). When we added each of the health insurance factors in Model III, the medical aid beneficiaries were 1.23 times more likely to receive outpatient care than those who were covered by NHI, after controlling for covariates (95 % CI: 1.20–1.26). When we added the interaction terms of the NHI and PHI factors in Model IV, those who were covered by both the medical aid program and PHI were 1.25 times more likely to receive outpatient care than those who were covered by only NHI (95 % CI: 1.21–1.29). In common with the utilization of inpatient care, those who were covered by only the medical aid showed a tendency to use fewer services than those who were covered by both the medical aid program and PHI, and they were 1.23 times more likely to receive outpatient care than those who were covered by only NHI (95 % CI: 1.19–1.26).

**Table 3:** Incidence risk ratio (IRR) and 95 % confidence interval (CI) of inpatient care utilization with four types of status of public and private health insurance in the Korea Health Panel Survey, 2008–2011

	Model I		Model II		Model III		Model IV	
	IRR	95 % CI	IRR	95 % CI	IRR	95 % CI	IRR	95 % CI
Gender (Ref.: Men)	1		1		1		1	
Women	1.08*	1.01–1.16	1.05	0.97–1.12	1.07*	1.00–1.15	1.07*	1.00–1.15
Age (Ref.: 20–29)	1		1		1		1	
30–39	0.81**	0.70–0.95	0.78**	0.67–0.91	0.76**	0.65–0.89	0.76**	0.65–0.89
40–49	0.67**	0.57–0.79	0.60**	0.51–0.71	0.58**	0.49–0.68	0.58**	0.49–0.68
50–59	0.85	0.72–1.01	0.73**	0.61–0.88	0.73**	0.61–0.87	0.73**	0.61–0.87
60 or older	1.31***	1.11–1.55	1.05	0.87–1.26	1.05	0.87–1.26	1.05	0.87–1.26
Chronic disease (Ref.: No)	1		1		1		1	
Yes	2.31***	2.14–2.49	2.27***	2.11–2.45	2.18***	2.03–2.36	2.19***	2.03–2.36
Marital status (Ref.: Single)	1		1		1		1	
Married couple	1.99***	1.72–2.31	1.98***	1.71–2.89	2.08***	1.80–2.41	2.08***	1.80–2.41
Divorced or Separated	2.55***	2.05–3.17	2.40***	1.93–2.98	2.25***	1.81–2.80	2.25***	1.81–2.80
Separation by death	2.00***	1.66–2.41	1.91***	1.58–2.30	1.91***	1.59–2.31	1.91***	1.58–2.30
Education (Ref.: College Degree or higher)			1		1		1	
High School to associate			1.23***	1.12–1.35	1.20***	1.10–1.32	1.20***	1.10–1.32
Middle School to associate			1.26***	1.11–1.43	1.19**	1.05–1.35	1.19**	1.05–1.35
Elementary School or less			1.36***	1.21–1.54	1.31***	1.16–1.48	1.31***	1.16–1.48
Household income (Ref.: 1^st^ quartile, lowest)			1		1		1	
2^st^ quartile			1.06	0.98–1.15	1.06	0.98–1.15	1.06	0.98–1.15
3^st^ quartile			1.09*	1.01–1.18	1.07	0.99–1.17	1.07	0.99–1.17
4^st^ quartile (highest)			1.15**	1.06–1.26	1.07	0.98–1.16	1.07	0.98–1.17
Healthcare covered by NHI (Ref.: Yes)					1			
No (medical aid)					2.26***	2.01–2.53		
Purchase of PHI (Ref.: No)					1			
Yes					1.06	0.98–1.15		
NHI status x PHI status (Ref.: Covered by only NHI)							1	
Covered by only the medical aid							2.37***	2.00–2.75
Covered by both NHI and PHI							1.08	0.99–1.17
Covered by both the medical aid and PHI							2.38***	1.97–2.76

*Note*: *NHI* National Health Insurance, *PHI* Private Health Insurance, Dependent variable: used inpatient care during the last year (1); not used inpatient care during the last year (0)

All models are additionally adjusted for age, gender, marital status, and chronic diseases

*p < 0.05

**p < 0.01

***p < 0.001

**Table 4:** Incidence risk ratio (IRR) and 95 % confidence interval (CI) of outpatient care utilization with four types of status of public and private health insurance in the Korea Health Panel Survey, 2008–2011

	Model I		Model II		Model III		Model IV	
	IRR	95 % CI	IRR	95 % CI	IRR	95 % CI	IRR	95 % CI
Gender (Ref.: Men)	1		1		1		1	
Women	1.58***	1.53–1.63	1.52***	1.47–1.57	1.52***	1.47–1.57	1.52***	1.48–1.57
Age (Ref.: 20–29)	1		1		1		1	
30–39	1.09***	1.05–1.13	1.05**	1.01–1.09	1.04*	1.01–1.09	1.05*	1.01–1.09
40–49	1.29***	1.24–1.35	1.20***	1.15–1.25	1.19***	1.14–1.24	1.19***	1.14–1.24
50–59	1.73***	1.65–1.81	1.52***	1.45–1.59	1.51***	1.44–1.58	1.51***	1.44–1.58
60 or older	2.40***	2.29–2.51	2.04***	1.94–2.14	2.03***	1.93–2.13	2.03***	1.93–2.13
Chronic disease (Ref.: No)	1		1		1		1	
Yes	1.98***	1.96–2.01	1.97***	1.94–2.00	1.97***	1.94–1.99	1.97***	1.94–1.99
Marital status (Ref.: Single)	1		1		1		1	
Married couple	1.73***	1.66–1.81	1.67***	1.60–1.75	1.69***	1.61–1.79	1.69***	1.61–1.76
Divorced or Separated	1.95***	1.84–2.07	1.86***	1.75–1.98	1.85***	1.74–1.97	1.85***	1.74–1.97
Separation by death	1.92***	1.82–2.02	1.76***	1.70–1.89	1.79***	1.70–1.89	1.79***	1.70–1.89
Education (Ref.: College Degree or higher)			1		1		1	
High School to associate			1.16***	1.12–1.21	1.15***	1.11–1.19	1.15***	1.11–1.19
Middle School to associate			1.34***	1.28–1.41	1.33***	1.27–1.39	1.33***	1.27–1.39
Elementary School or less			1.55***	1.48–1.63	1.53***	1.46–1.60	1.53***	1.46–1.60
Household income (Ref.: 1^st^ quartile, lowest)			1		1		1	1
2^st^ quartile			0.98**	0.97–0.99	0.98**	0.97–0.99	0.98**	0.97–0.99
3^st^ quartile			0.99**	0.97–0.99	0.99	0.98–1.00	0.99	0.98–1.00
4^st^ quartile (highest)			0.99	0.98–1.00	0.99	0.98–1.00	0.99	0.98–1.00
Healthcare covered by NHI (Ref.: Yes)					1			
No (medical aid)					1.23***	1.20–1.26		
Purchase of PHI (Ref.: No)					1			
Yes					1.02	0.99–1.03		
NHI status x PHI status (Ref.: Covered by only NHI)							1	
Covered by only the medical aid							1.23***	1.19–1.26
Covered by both NHI and PHI							1.01	0.99–1.03
Covered by both the medical aid and PHI							1.25***	1.21–1.29

*Note NHI* National Health Insurance, *PHI* Private Health Insurance, Dependent variable: used outpatient care during the last year (1); not used outpatient care during the last year (0)

All models are additionally adjusted for age, gender, marital status, and chronic diseases

*p < 0.05

**p < 0.01

***p < 0.001

## Discussion

User fees have increased substantially in many developed countries during the last 20 years [21, 22, 32]. The hasty introduction of a PHI program can lower essential healthcare utilization by those who are economically disadvantaged, while the advantages of the adoption of PHI are relatively modest [33]. In the case of Korea, however, there is a policy of medical aid, with a ceiling system implemented along with a co-payment system, which lessens the likelihood that poor individuals will not have access to essential healthcare [21]. Rather, the moral hazard phenomenon is becoming worse. In fact, we found that healthcare utilization by the group with medical aid who had more covered healthcare service benefits was the highest compared to that of the group with only NHI after controlling for their health status. This could cause a crisis in NHI, which is based on social solidarity and risk pooling. We therefore examined the effects of public and private health insurance on medical service utilization in the health security system of Korea. We also investigated possible instances of moral hazard among individuals with PHI under Korea’s universal health care system.

First, the medical aid beneficiaries who were offered nearly free medical services were more likely to receive inpatient and outpatient care than those who were covered by only NHI. These results are consistent with the findings of other studies, which reported that health insurance coverage may cause a reduction in prevention activities [15, 16]. There are also cost-sharing effects which encourage moral hazard–induced utilization [19–21]. The growth of the utilization of medical services due to the expansion of the covered population could cause distortions in the healthcare delivery system. The method of supplementing the coverage of NHI, as it exists in Korea, could thus weaken primary healthcare services and therefore reduce the need for inpatient care through preventive services, while also increasing household medical expenses [34, 35]. When the management of the insurance system is not clear, especially for vulnerable groups, the public healthcare security system can be detrimentally affected.

Second, although those who were covered by only PHI had no significantly effect on medical utilization, there was a positive correlation between these two variables. Moreover, according to an analysis of the interaction effect, those who were covered by both the medical aid program and PHI were more likely to receive inpatient and outpatient care than those who were covered by NHI. This is analogous to results presented in other reports [27, 30]. In those studies, the probability of health care utilization was found to be higher for when people have more PHI. The role of PHI under NHI is usually to provide services which cannot be included in the coverage given to the public sector and to improve the quality of services [18]. However, it could eventually increase not only household medical expenses but also total government spending on health care [6]. Another crucial problem is the possibility that the middle and upper income groups are able to receive more medical services through PHI, whereas socially vulnerable groups have difficulty obtaining medical services despite their greater need. Those who enrolled in PHI under the NHI system are usually people of high socioeconomic status [24]. If this form of private insurance can serve as a substitute for PHI among the types of PHI, it would contribute to strengthening health equality. However, a fringe benefit of supplementary PHI in Korea is that it is strongly focused on commerciality, which could worsen the health disparities between the insured and non-insured and damage social solidarity. For example, the supplementary PHI of France eventually worsened the degree of inequality in healthcare utilization, and the portion of public expenditures on national medical expenses was not reduced [25]. Thus, policies for managing the borderline between PHI and NHI more effectively and for securing the availability of health services socially are needed to reduce health inequality or disparities [36].

Nevertheless, several limitations of this study should be noted. First, the severity of disease can affect the quantity of healthcare utilization, but such a relationship was not reflected in the model. However, this study shows that the group with PHI has a greater possibility of receiving outpatient care as well as inpatient care, and the amounts are even higher for the group with the medical aid. In the United States, given that for any health problem only 8 to 9 out of 1,000 people in a local community receive inpatient care, the phenomenon in which the increased cost of primary healthcare services is related to the increase in inpatient care can be considered as an outcome of moral hazard [37]. Nevertheless, medical suppliers, including the physician-induced demand phenomenon, make it necessary to emphasize relevant information about medical service utilization in the model. Second, the health outcomes of the respondents after they had received medical services were not reflected in the model, implying that the effects of the insurance on the patient health outcomes are unclear [38].

## Conclusion

Healthcare utilization including inpatient and outpatient care is critically determined by each patient’s behavior before a doctor clinically determines what is causing the patient’s symptoms. The significant finding of this study is that differences in the quantity of healthcare utilization are definitely related to whether a patient uses NHI or PHI. This result reveals that expanding insurance coverage is crucial for inducing the use of medical care after adjusting for health status confounders. Hence, the social problem of “moral hazard can arise” with regard to insurance membership depending on how the NHIS maintains policies to confer effective benefits to patients, despite the fact that it is difficult to predetermine what amount of services is appropriate for a patient given their unique situations. Moreover, the moral hazard phenomenon may give rise to differences of medical utilization while also resulting in a financial burden due to increasing overall medical costs in South Korea. Hence, from this work, which considers the access and use of medical services under different insurance types, policymakers can obtain helpful information when reforming the health care delivery system. Furthermore, they must scrutinize the insurance system to determine how best to reform NHI in order to solidify the rights of medical service consumers through PHI.

## Abbreviations

KHP: Korea health panel
NHI: National health insurance
NHIS: NHI service
PHI: Private health insurance
